# Wideband, Wearable, Printed Monopole Antenna System for Integration into an Electromagnetic Radiation Evaluation Device

**DOI:** 10.3390/s23073667

**Published:** 2023-03-31

**Authors:** Pedro Falcão, João M. Felício, Custódio Peixeiro

**Affiliations:** 1 Instituto de Telecomunicações, Instituto Superior Técnico, University of Lisbon, Av. Rovisco Pais, 1049-001 Lisbon, Portugal; 2 Escola Naval, Instituto Universitário Militar, Navy Research Center, Base Naval de Lisboa—Alfeite, 2810-001 Almada, Portugal

**Keywords:** wearable antenna, wideband antenna, planar printed monopole, dual-linearly polarized antenna, electromagnetic field evaluation

## Abstract

This paper describes the design steps carried out to prove the concept of a wideband monopole antenna system to be used in a wearable device conceived for the evaluation of electromagnetic field radiation. Such a device is envisaged to be integrated into protective vests worn by professional users in their working space environment as part of intelligent multi-risk protection. Initially, the main characteristics of a simple straight monopole are reviewed to serve as a reference. A modified octagonal monopole antenna element is introduced, and a two dual-linearly polarized configuration of such monopoles is designed, fabricated, and tested to be used in the frequency range of 0.7–3.5 GHz. The expected radiation characteristics (input reflection coefficient and isolation between vertically and horizontally polarized ports) are confirmed experimentally. The effects of a thick lossy foam substrate layer used to mitigate the presence of the metal shield, employed in the vest lining as a Faraday cage protection, are analyzed both by simulation and experimentally. Finally, electromagnetic simulations are carried out to confirm that a system of five dual-linearly polarized monopole elements located in the chest, shoulders, back, and helmet of the user can provide an adequate estimation of the incident electromagnetic field radiation.

## 1. Introduction

In the last thirty years, there has been a remarkable increase in the deployment of mobile communication and Wi-Fi systems. Notably, 2G, 3G, and 4G standards represent a step forward in such an increase. With the advent of 5G, an enormous boost in the number of users and terminals, as well as in traffic, is expected [1]. With the densification of the networks, telecommunication manufacturers and operators need to take effective precautions to protect their staff from radiation hazards [2].

This paper presents the proof-of-concept for the development of an antenna system envisaged to be integrated into protective vests worn by professional users in their working space environment as part of intelligent multi-risk protection [3]. Such a system will measure and display the amplitude of the impinging electromagnetic field in the frequency range of 0.7–3.5 GHz. This frequency range contains all current mobile communication systems standards, that is, 2G, 3G, 4G, and 5G (below 6 GHz).

The proof-of-concept for the development of the antenna system is divided into three steps. The first step corresponds to the design and optimization of the basic antenna element, a wideband dual-linearly polarized monopole, in free space [4]. In the second step, the evaluation of the influence of the lossy foam used to mitigate the unwanted effects of the protective metal shield that covers the inner part of the vest, in the monopole characteristics, is carried out [5]. The third and last step corresponds to the evaluation of the monopole antenna system integrated into the vest. A simplified model of the user’s body is chosen to be compliant with future measurements and practical fabrication constraints. Numerical simulation results of the induced voltage in each antenna port allow the validation of the estimation of the amplitude of the impinging electromagnetic field.

It is important to highlight the following innovative features proposed and described in this paper:

- The modification was introduced in the octagonal printed planar monopole antenna element to further meander the current distribution.
- The compact combination of two linearly polarized monopoles was used to obtain dual-linear polarization, with acceptable mutual coupling.
- A foam absorber layer was used to mitigate the unwanted effects of a metal shield.
- The configuration of the antenna system was used to provide full coverage of the environment around the user.
- A method was used to estimate the amplitude of the electric field of the incident electromagnetic plane wave.

The paper is organized into five sections. After this introduction (Section 1), Section 2 describes the design of the basic dual-linearly polarized monopole antenna element. A detailed analysis of the simple straight monopole is included as a reference in this section. Section 3 deals with the insertion of an absorbing foam layer to mitigate the unwanted effects of the metal shield. Section 4 presents the final antenna system and describes the estimation of the impinging electromagnetic field. Finally, Section 5 contains the main conclusions and discusses the future work needed to evolve from the proof-of-concept provided to the final prototype.

## 2. Monopole Antenna Element

The dipole antenna has been used since the 1887 Heinrich Hertz founding experiment to prove the existence of electromagnetic waves predicted by James Clerk Maxwell [6]. It is one of the most successful types of antennas and can be regarded as two balanced fed monopoles. The monopole is a modification of the dipole, where the bottom monopole is replaced by a ground plane. Both dipoles and monopoles can either be made of wire or printed in planar PCB structures. The latter benefits from the well-known advantages of PCB technology, such as low cost, low profile, compactness, and reproducibility and is used typically above VHF and mostly at microwave frequencies [7].

### 2.1. Planar Straight Monopole

**Figure 1:**
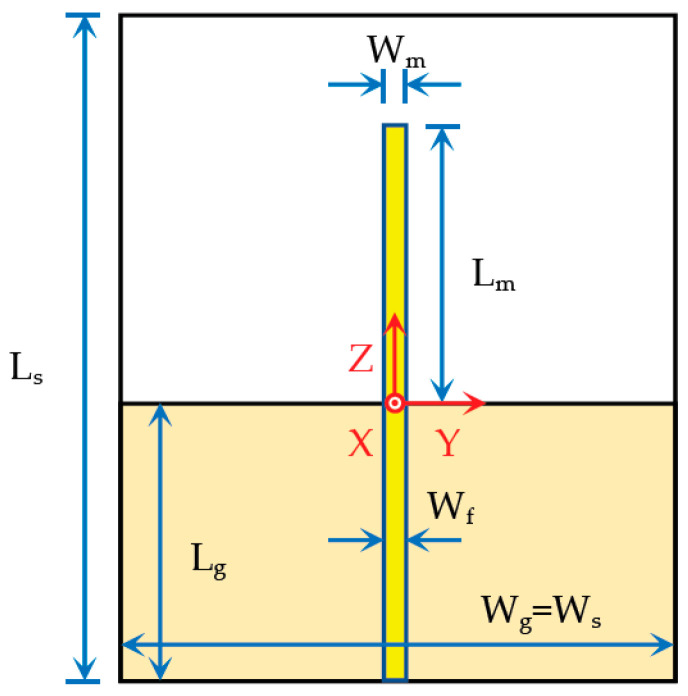
Printed planar straight monopole configuration.

The simplest printed monopole antenna configuration is shown in Figure 1.

It is composed of a straight metal strip printed on one side of the substrate and the ground plane printed on the other side. This simple monopole will not fulfill the impedance bandwidth specifications (0.7–3.5 GHz). It is presented and analyzed here just to serve as a reference.

**Figure 2:**
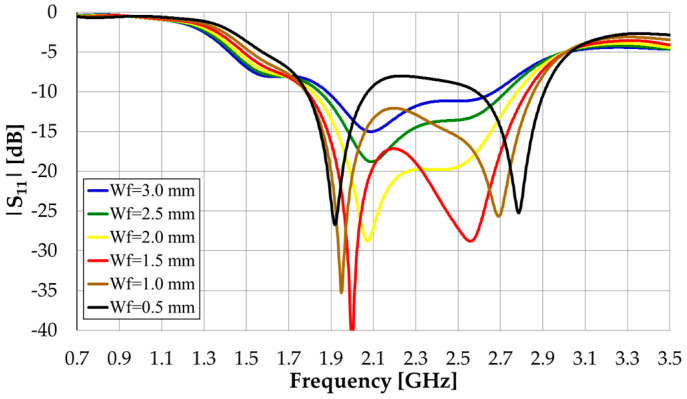
CST simulated results of the straight monopole input reflection coefficient amplitude.

Initially, the following dimensions have been used: L_s_ = W_s_ = W_g_ = 150 mm, L_g_ = 50 mm, and W_m_ = W_f_ = 3 mm. A cheap 1.6 mm thick FR4 substrate with relative permittivity ε_r_ = 4.3 and loss tangent tanδ = 0.025 was chosen. The monopole length was optimized to have the first resonance at the central frequency (f_0_ = 2.1 GHz) of the working frequency band 0.7–3.5 GHz. L_m_ = 25 mm = 0.175λ_0_ was obtained. The microstrip feed line width was swept from 3.0 mm to 0.5 mm with a 0.5 mm step. This corresponds to characteristic impedances of the microstrip feed line ranging from 50.7 Ω up to 110.6 Ω [8]. The input reflection coefficient results obtained with the time domain solver of the CST Studio Suite software package [9] are shown in Figure 2. A 50 Ω SMA coaxial connector, at the end of the microstrip feed line, has been included in the CST model.

We define the impedance bandwidth using the usual |S_11_| = −10 dB reference level, which corresponds to a 10% reflected power level. With this criterion, the larger bandwidth is obtained for W_f_ = 1.0 mm and is about 46% (1.78–2.86 GHz), far from the required 133.3% or 5:1 (0.7–3.5 GHz), as expected.

**Figure 3:**
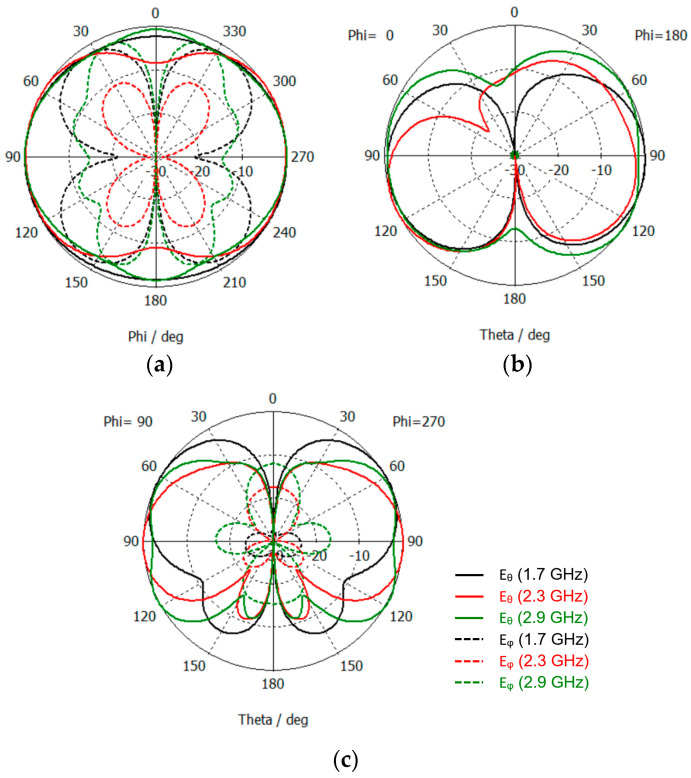
CST simulated far-field 2D radiation pattern results of the straight monopole. (**a**) H-plane (XY); (**b**) E-plane (XZ); (**c**) E-plane (YZ).

**Figure 4:**
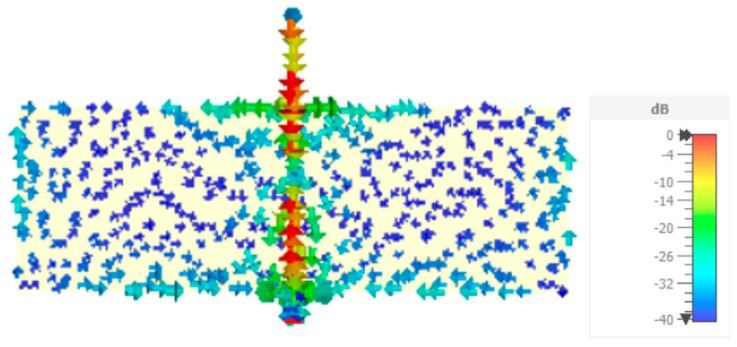
CST simulated current distribution of the straight monopole configuration at 2.3 GHz.

The far-field radiation pattern has also been simulated in CST, for the W_f_ = 1.0 mm configuration. The results obtained for the 2D cuts in the principal planes are shown in Figure 3. As it can be verified, the radiation pattern is almost omnidirectional in the whole frequency range of interest. Moreover, even in the E-planes, despite the electrical length of the monopole ranges from 0.142λ (at 1.7 GHz) up to 0.242λ (at 2.9 GHz) the radiation pattern does not change much. For the monopole orientation chosen, not only the E_θ_ polarization component is meaningful. Especially in the H-plane, for 1.7 GHz and 2.9 GHz, the amplitude of the E_φ_ component is similar to the E_θ_ one, for φ ≈ 30° (and, due to physical symmetry, 150°, 210°, and 330°). This is a specificity of the planar monopole that can be explained by the current distribution in the ground plane (shown in Figure 4). In the case of the classical non-planar monopole, where the ground plane is perpendicular to the monopole, the E_φ_ component is canceled everywhere due to the perfect cylindrical symmetry of the structure [10].

**Figure 5:**
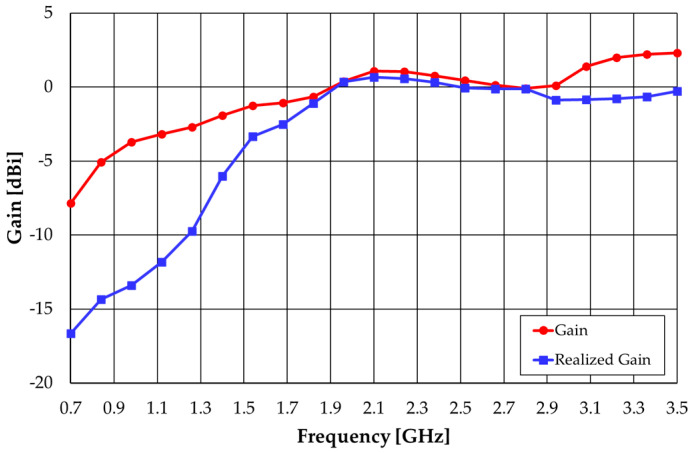
CST simulated gain results of the straight monopole.

The gain results, shown in Figure 5, also express a relatively stable value in the frequency range of 1.7 GHz to 2.9 GHz. The realized gain takes into account the input impedance mismatch [11].

The current distribution on the straight monopole configuration, at 2.3 GHz, is shown in Figure 4. As can be seen, the current is stronger along the monopole, and the feed line, vertical strip. However, the current in the ground plane is also meaningful and also has a horizontal component. This horizontal component is particularly intense in the horizontal edges of the ground plane, near the vertical strip of the monopole and feed line, which explains the magnitude of the E_φ_ component in the XY plane, as mentioned before.

**Figure 6:**
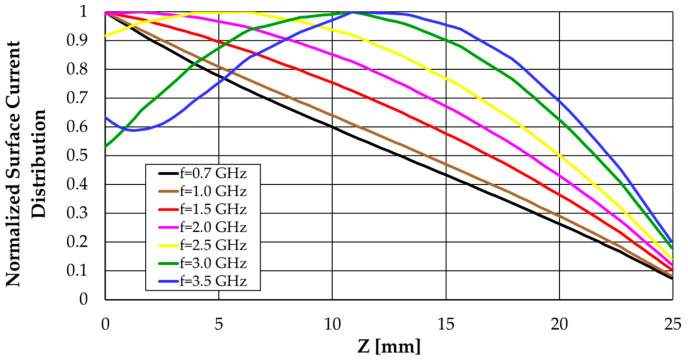
CST simulated vertical surface current distribution along the straight monopole.

The CST simulation results of the Z-component of the surface current distribution along the monopole are shown in Figure 6. These results agree with the sinusoidal distribution approximation of the thin wire dipole/monopole [10]. Moreover, the current at the end of the monopole (Z = 25 mm) does not vanish due to the fringing effect, which is more effective as frequency increases.

### 2.2. Modified Octagonal Monopole

Many techniques have been used to improve the impedance bandwidth of printed planar monopoles [12,13,14,15,16,17,18,19,20,21,22,23,24,25,26,27,28,29,30,31,32,33,34,35,36,37,38,39]. Considering the evolution from the simple straight monopole analyzed in the previous sub-section, these techniques can be grouped into the following four categories:

- Change the geometry of the monopole.
- Change the geometry of the ground plane.
- Change the type of feeding.
- Change the types of materials and/or use active devices.

Many geometries have been proposed, with canonical shapes (square [13], triangular [14,15], circular [16], elliptical [17], hexagonal [18], and octagonal [19]), fractal shapes [20], bio-inspired shapes [21,22] and even more fancy shapes [23,24,25]. Many ground plane shapes have been used [18,24,26,27,28,29,30,31] as well as different types of feeding [18,26,28,31,32,33,34,35]. Moreover, different types of materials have been employed, such as substrate or superstrate layers [12,22,36,37,38] and/or active devices (mainly varactors [30], MEMS [33], and PIN diodes [39]). Many references combine two [13,14,18,25,27,36,39], three [19,22,24,26,28,29,30,31,33,34,37,38], or even four categories of change [12,35] to obtain a very wide bandwidth. For instance, in [28] a 25:1 bandwidth (1.08 to 27.4 GHz) is reported.

**Figure 7:**
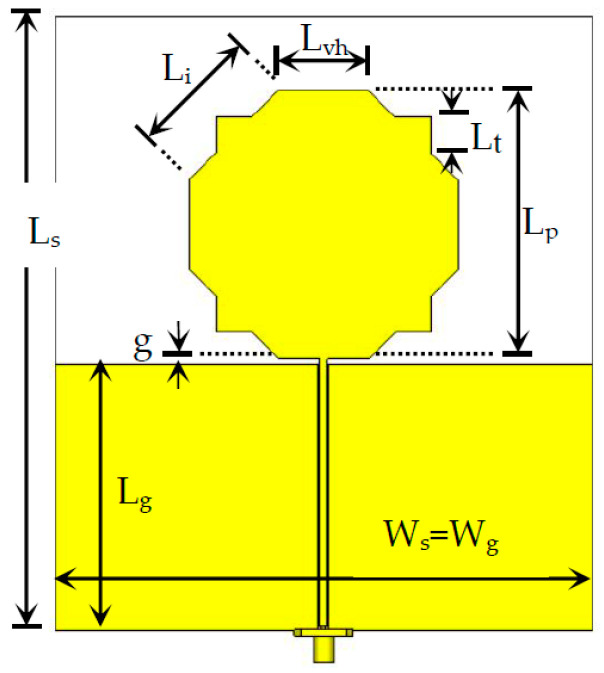
Modified octagonal monopole antenna element with CPW feed.

The selection of the monopole’s shape, the geometry of the ground plane, the type of feeding, and the type of materials depend mostly on the type of frequency response required, and the space available. For the intended application, where a single wideband (5:1) is specified, a simple configuration that meets the requirements was selected. Initially, the octagonal-shaped monopole [19,31] was selected as a good compromise between the space available and the specified frequency range of operation. However, it was concluded that the shape needed to be further meandered to fulfill the bandwidth specifications. Therefore, right triangles have been added to alternate sides of the octagon to keep the monopole high. Such triangles were not added at the top and bottom sides. The modified octagonal-shaped monopole shown in Figure 7 was then introduced [4].

A simple rectangular ground plane and a thin single-layer substrate configuration are used. Moreover, a coplanar waveguide (CPW) feed is utilized. The novelty of the proposed configuration corresponds to the use of right triangles on four edges of the octagonal patch to enhance meandering. The octagonal patch is not regular (L_i_ ≠ L_vh_) to increase flexibility in the design process. The values used for the geometrical parameters are indicated in Table 1. They have been adjusted, one by one, throughout a complete sensitivity analysis carried out with CST Studio Suite numerical simulations. Parameters a and b correspond to the widths of the CPW central strip and slots.

**Figure 8:**
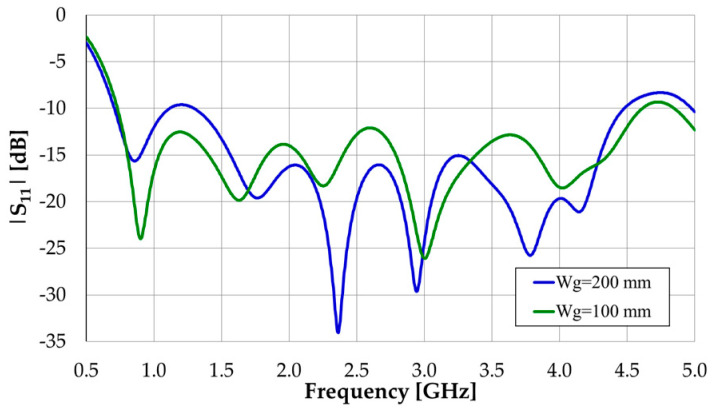
Input reflection coefficient amplitude simulation results of the modified octagonal monopole with CPW feed.

To check the effect of the ground plane size, simulations have also been carried out for the case W_s_ = W_g_ = 200 mm. The corresponding results are shown in Figure 8.

It can be concluded that for the two ground plane widths considered, the ground plane width has a small effect on |S_11_| simulation results. The results are slightly better for W_g_ = 100 mm (with |S_11_| < −10 dB in the frequency range of 0.73–4.61 GHz) because the other geometrical parameters have been optimized for that case. It can also be concluded that the design equation of the octagonal monopole applies, that is, the patch length is slightly less than a quarter of the free-space wavelength at the frequency of the first resonance (L_p_ = 0.225λ, at 0.90 GHz, for W_g_ = 100 mm) [19,31].

**Figure 9:**
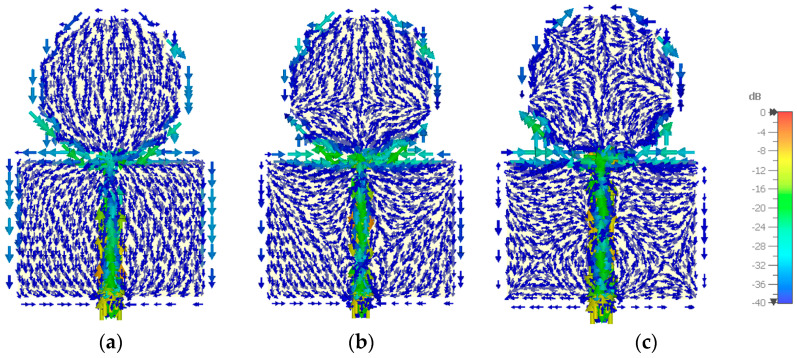
CST simulated normalized current distribution on the modified octagonal monopole configuration. (**a**) 0.7 GHz; (**b**) 2.1 GHz; (**c**) 3.5 GHz.

The simulated current distribution on the modified octagonal monopole, at the limits and center of the frequency band of operation, is shown in Figure 9. As expected, the current distribution shows big changes in the frequency range of operation (5:1). For the three frequencies shown, the monopole and ground plane electric lengths are 0.175, 0.525, 0.875, 0.233, 0.700, and 1.167, respectively. At 0.7 GHz, the current is mainly vertical and pointing downwards, whereas at 2.1 GHz, it is also mostly vertical and pointing downwards in the initial two-thirds of the monopole and mostly horizontal in the last third, with the vertical component pointing upwards. At 3.5 GHz, the current distribution is much more complex, with several changes both in the vertical and horizontal components. This complexity can be explained in terms of the higher-order modes established by the theory of characteristic modes [40,41].

**Figure 10:**
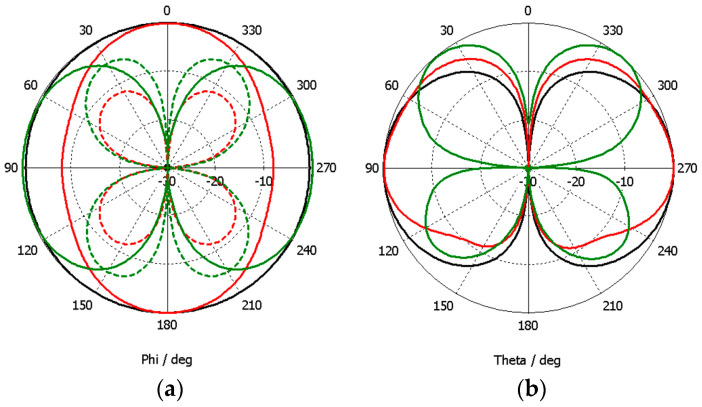

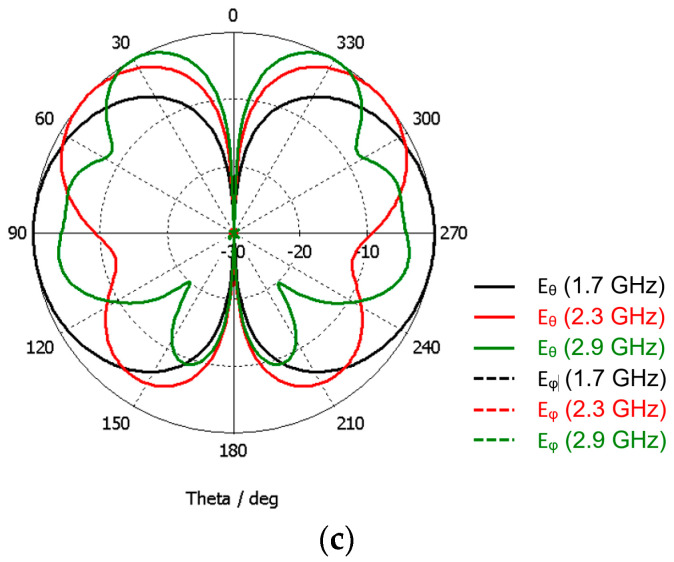
CST simulated far-field 2D radiation pattern results of the modified octagonal monopole. (**a**) H-plane (XY); (**b**) E-plane (XZ); (**c**) E-plane (YZ).

The simulated far-field 2D radiation pattern results are shown in Figure 10.

The YZ plane radiation pattern does not change much in the whole frequency range of operation. However, on the other two principal planes, the radiation pattern is quite different at 3.5 GHz. This difference can be justified by the existence of the higher-order modes already referred to above [40,41]. The cross-polarization component behaves the same way as in the simple straight monopole.

**Figure 11:**
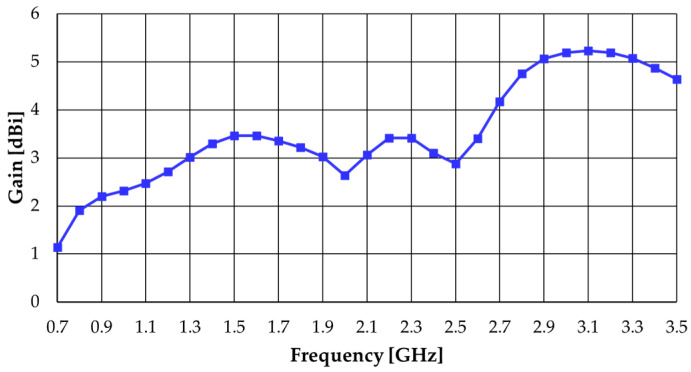
CST simulated realized gain results of the modified octagonal monopole.

The gain simulated results are shown in Figure 11. The realized gain tends to increase as frequency increases and spans the range of 1.1–5.2 dBi with an average of 3.5 dBi.

### 2.3. Dual-Linearly Polarized Monopole

**Figure 12:**
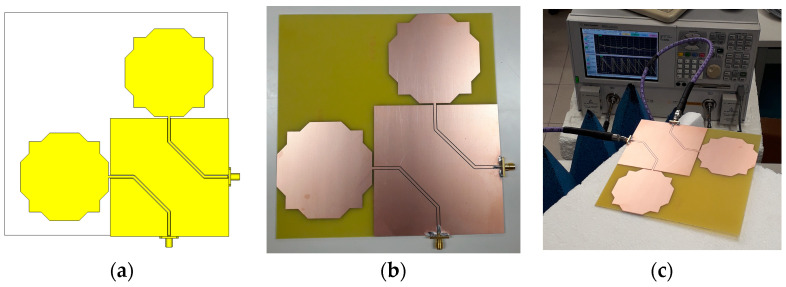
Dual-linearly polarized modified octagonal monopole antenna configuration. (**a**) Geometry; (**b**) Prototype; (**c**) Measurement of S-parameters with a VNA.

To avoid the possibility of a complete polarization mismatch, two modified octagonal monopoles with orthogonal orientations were combined, as shown in Figure 12. The sides of the square substrate and ground plane have 190 mm and 100 mm, respectively. The other geometrical parameters keep the values indicated in Table 1. The geometry of the CPW feeds has been chosen to minimize mutual coupling in a symmetrical arrangement. The distance between the two CPW lines is 35.4 mm.

**Figure 13:**
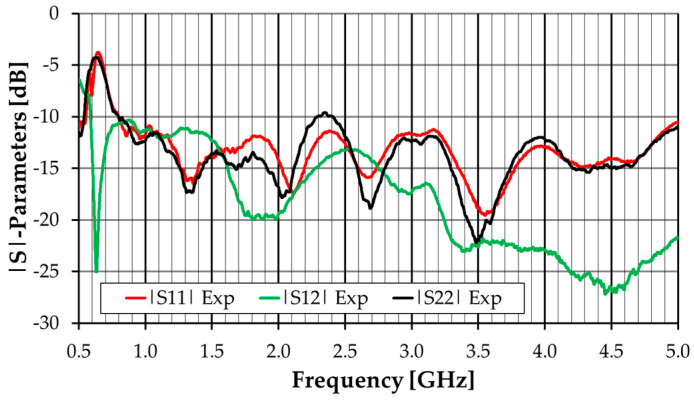
|S|-parameters experimental results of the dual-linearly polarized monopole antenna.

The amplitude of the experimental S-parameters is shown in Figure 13. For the sake of clarity, the simulation results are not shown, as they are very similar to these experimental ones. The mutual coupling is below −10.4 dB. Due to minor fabrication inaccuracies, the input reflection coefficient of the two ports is not the same, but it is quite identical. Both exceed the −10 dB reference level at the low end of the frequency range of operation. These relatively high values of the input reflection coefficient and mutual coupling are mostly caused by the excessive truncation of the substrate and will be fixed in the next stage of the antenna development (Section 3.2).

## 3. Dual-Linearly Polarized Monopole Antenna with Absorber and Metal Shield

This section deals with the dual-linearly polarized monopole antenna element in the presence of a metal shield (with corresponds to the protective metal lining of the vest). Lossy foam is placed between the substrate, the ground plane, and the metal shield to absorb the reflections. First, the foam material is macroscopically characterized experimentally, and then the foam layer thickness is optimized.

### 3.1. Experimental Characterization of the Foam Absorber

The absorber layer was obtained by cutting slices of the planar region of an anechoic chamber absorber panel. As the macroscopic characteristics (ε, μ, σ) of the panel material are not homogenous, they need to be measured. However, the foam has normal magnetic behavior (μ = μ_0_), and therefore only ε and σ need to be measured.

**Figure 14:**
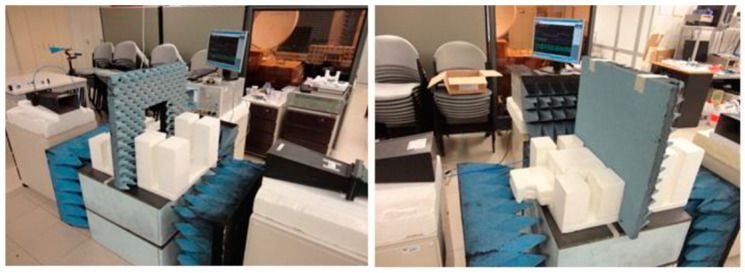
Setup for experimental characterization of the absorber foam.

The real and imaginary parts of the (complex) relative electric permittivity are defined as

(1) $$\varepsilon_{r}^{\prime }-j\;\varepsilon_{r}^{\prime \prime }=\varepsilon_{r}-j\;\frac{\sigma }{\omega \varepsilon_{0}}=\varepsilon_{r}\left(1-j\;tan\delta \right)$$

where ε_r_ is the relative electric permittivity, σ is the conductivity, ω is the angular frequency, and tanδ is the loss tangent. Both ε_r_′ and ε_r_″ are measured using the free-space transmission method [42], where the amplitude and phase of the transmission coefficient of a link between two horn antennas are measured in a VNA (Figure 14), with and without the absorber layer in the middle.

**Figure 15:**
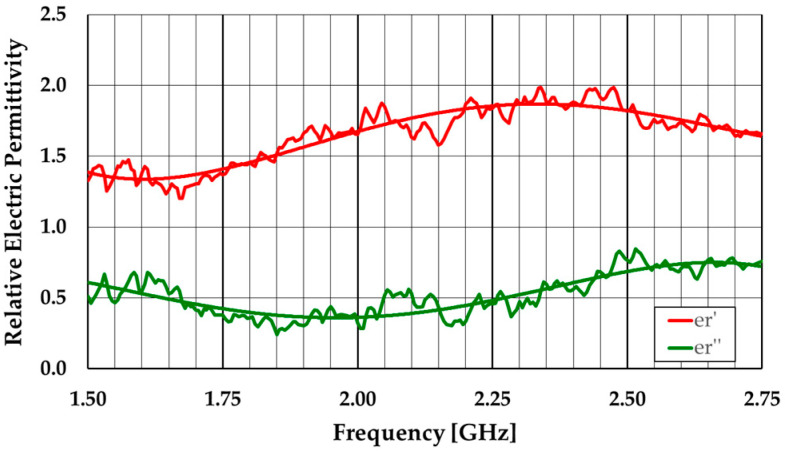
Example of experimental ε_r_′ and ε_r_″ results.

As an example, ε_r_′ and ε_r_″ experimental results, obtained for part of the frequency band of interest, are shown in Figure 15. These results have been used to calibrate the model of the absorber used in CST Studio Suite [9]. The ripple observed in the results is caused by reflections in the non-anechoic environment of the room.

### 3.2. Dual-Linearly Polarized Monopole Antenna Optimization and Test

**Figure 16:**
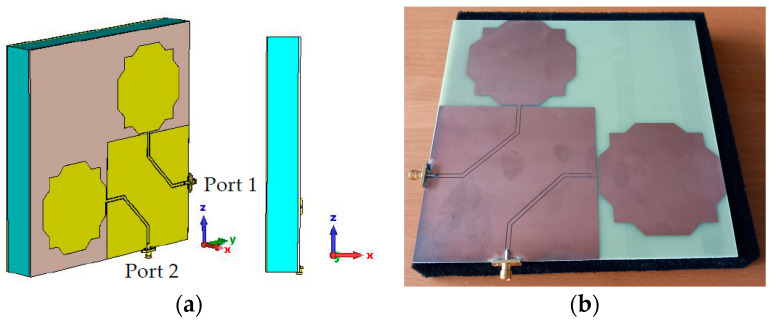
Final monopole antenna configuration. (**a**) CST model. (**b**) Photo of the prototype.

The dual-linearly polarized monopole antenna element with an absorbing foam layer and a metal shield is shown in Figure 16.

**Figure 17:**
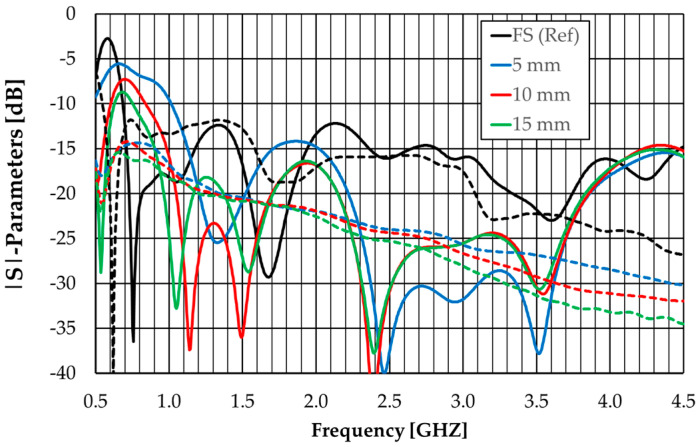
|S|-parameters’ simulation results of the modified octagonal monopole with absorber and metal shield, for different absorber thicknesses. |S_ii_|—solid lines, |S_12_|—dashed lines.

This antenna structure has been simulated using an absorbing layer with thicknesses of 5, 10, and 15 mm. The obtained amplitudes of the S-parameters are shown in Figure 17. The results of the free-space configuration are also included as references.

An absorber thickness of 10 mm provides |S_11_| (and |S_22_|) below −10 dB except in the range of 0.70–0.75 GHz where it is slightly above (−7.3 dB at 0.7 GHz). In addition, for an absorber thickness of 10 mm, the mutual coupling is below −14.2 dB.

**Figure 18:**
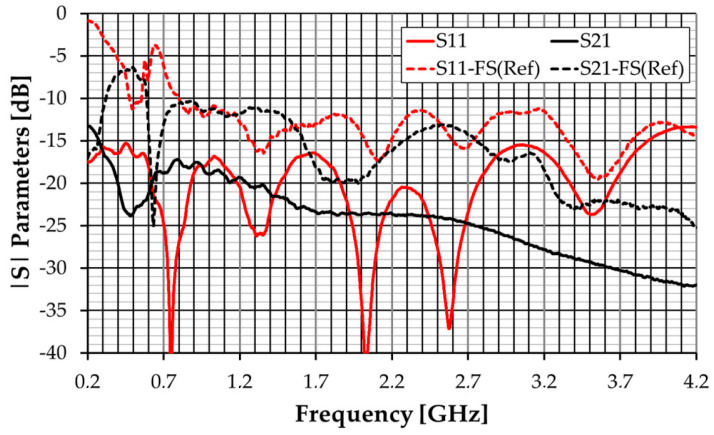
|S|-parameter experimental results of the modified octagonal monopole with absorber and metal shield.

An antenna prototype with a 10 mm thick absorber layer has been fabricated and tested. The corresponding |S_11_| and |S_21_| experimental results are shown in Figure 18. The S_22_ results (not included) generally show a good agreement with S_11_. The experimental results of the free-space prototype are also shown for reference. It can be verified that |S_11_| < −15.4 dB, and |S_21_| < −17.1 dB for the whole frequency band of operation. These results are substantially below (about 5 dB on average) the simulated one, especially at the lower frequencies. This is due to the less accurate characterization of the absorber in that frequency region as a pair of horn antennas is not available for such a frequency band. It can be concluded that it is feasible to use an absorber layer a few millimeters thinner.

**Figure 19:**
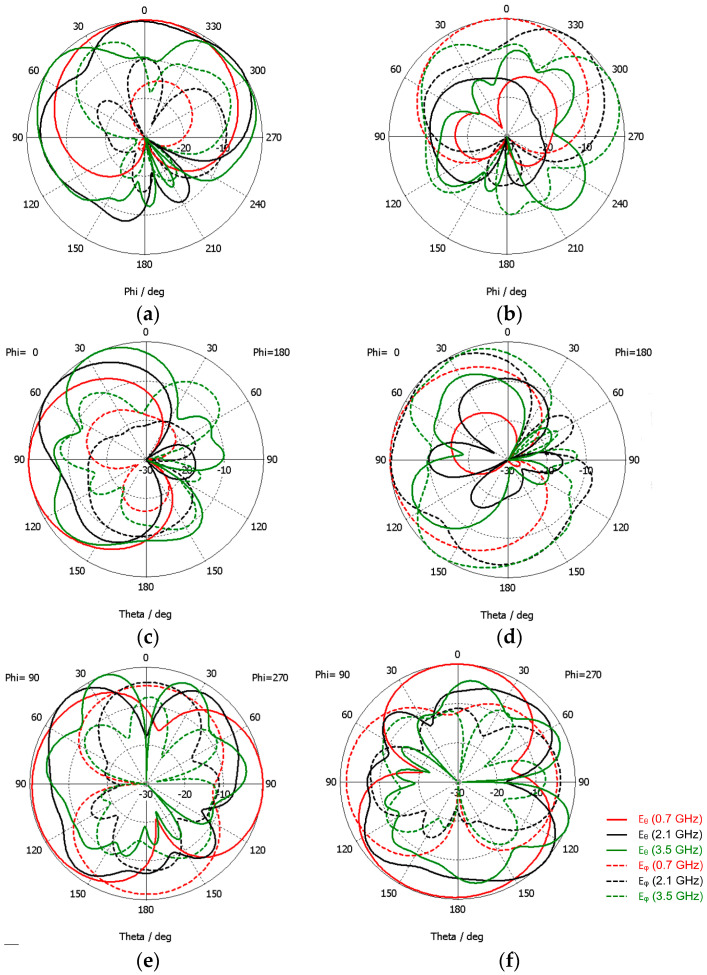
CST simulated far-field 2D radiation pattern results of the modified octagonal monopole with absorber and metal shield. (**a**) XY plane, Port 1; (**b**) XY plane, Port 2; (**c**) XZ plane, Port 1; (**d**) XZ plane, Port 2; (**e**) YZ plane, Port 1; (**f**) YZ plane, Port 2.

The simulated radiation pattern results for each port are shown in Figure 19.

As intended, the metal shield almost blocks the radiation to its back side. There are several physical symmetries reflected in the radiation pattern. For instance, the theta component of Port 1 in the XY plane corresponds to the phi component of Port 2 in the XZ plane, rotated 90 degrees, and vice versa. In the three principal planes, the radiation intensity level in the front hemisphere, for both polarizations, is below—5 dB in only one-third of the 180-degree angular region. Then, it can be concluded that the radiation pattern does not change much in most of the front hemispheres.

**Figure 20:**
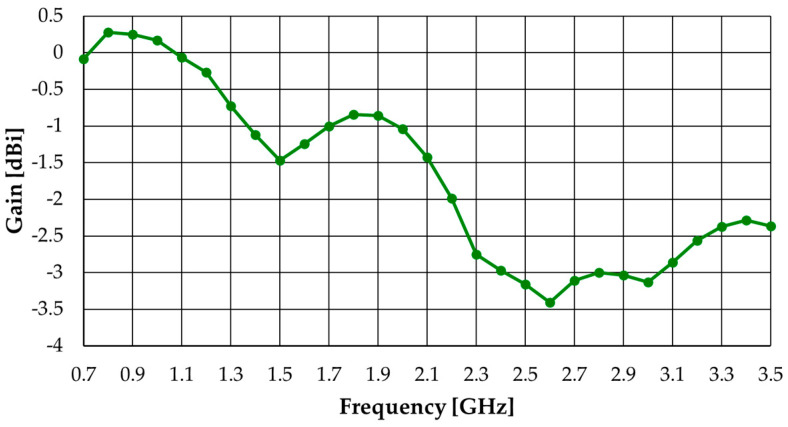
Simulated realized gain results of the modified octagonal monopole with absorber and metal shield.

**Figure 21:**
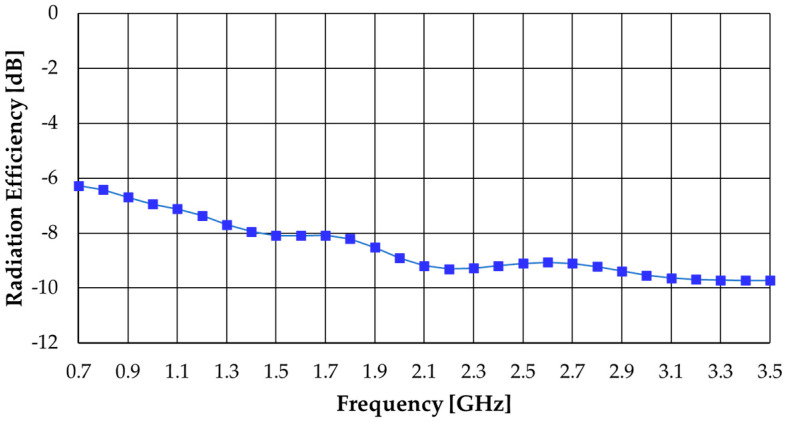
Simulated radiation efficiency results of the modified octagonal monopole with absorber and metal shield.

The corresponding realized gain and radiation efficiency simulated results are shown in Figure 20 and Figure 21, respectively. Due to the physical symmetry of the configuration, the two ports have the same gain and efficiency.

The gain spans the range of −3.4 to 0.3 dBi with an average of −1.7 dBi. Compared with the gain of the free-space monopole (shown in Figure 11) there is an average decrease of 5.2 dB, caused by the absorber layer.

The radiation efficiency decreases as frequency increases and spans the range of −9.9 dB (10.7%) at 3.5 GHz to −6.3 dB (23.6%) at 0.7 GHz. The decrease in efficiency is a natural consequence of the insertion of the absorber layer, a price to pay for the mitigation of the presence of the metal shield.

## 4. Final Antenna System Configuration

The dual-linearly polarized monopole antenna element, analyzed in the previous section, is used as the basic antenna element of the envisaged antenna system used to evaluate the incident electromagnetic field.

### 4.1. Monopole Antenna System Geometry

**Figure 22:**
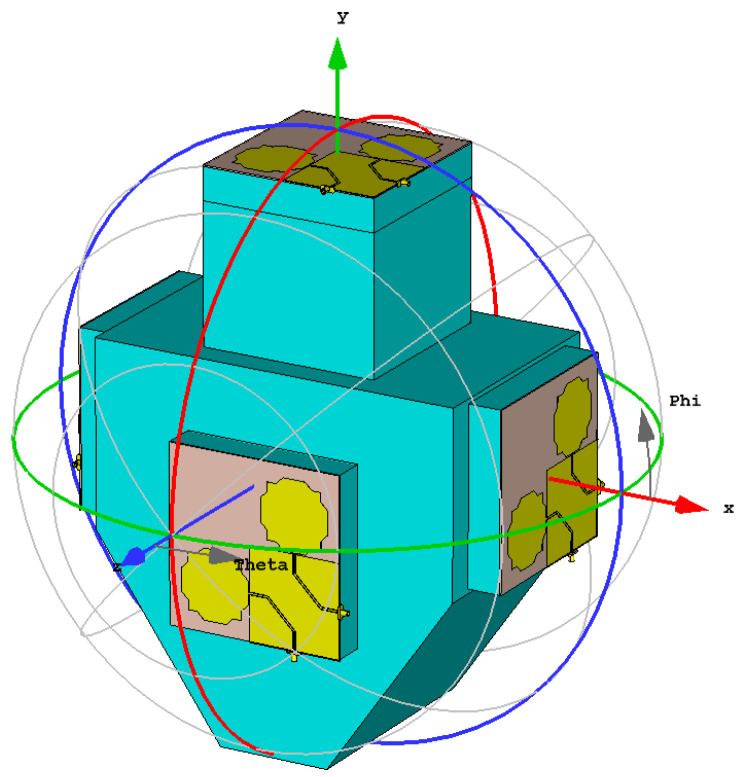
Proposed antenna system configuration.

The antenna system proposed for evaluating the incident electromagnetic field is shown in Figure 22.

Five dual-linearly polarized monopole antenna elements are spatially distributed to provide almost uniform coverage of the space around the user. Four such antennas are located on the torso (chest, right shoulder, back, and left shoulder), and the remaining one is located on the helmet. The torso has maximal dimensions 40 cm × 40 cm× 25 cm This simple model of the user was chosen according to the facilities that will be used to fabricate the physical phantom [43] needed to perform the final experimental validation of the proposed antenna system.

### 4.2. Numerical Simulation Results

The proposed antenna system was simulated using CST Studio Suite. The input reflection coefficient and gain of each antenna port have been obtained. Moreover, in receive mode, the voltage induced in each antenna port is also obtained for different directions of incidence and polarizations of an incoming plane electromagnetic wave. The port numbering is indicated in Table 2.

**Figure 23:**
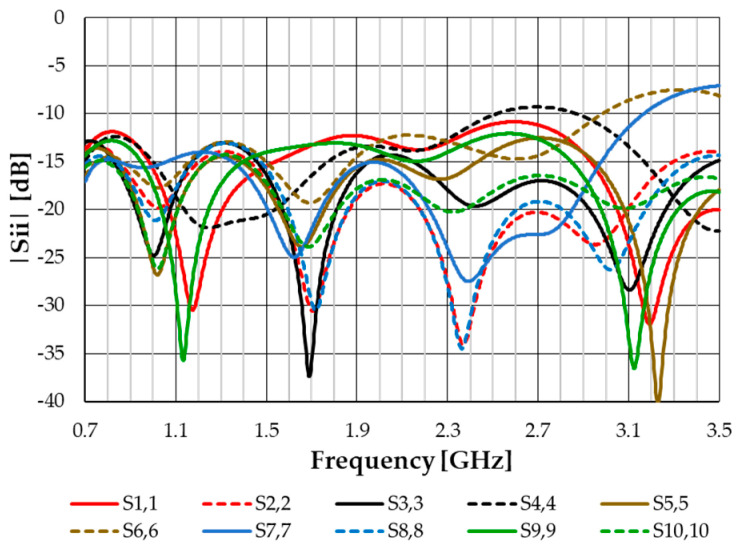
Simulated |S|-parameters’ results of the antenna system.

The simulated input reflection coefficient results are shown in Figure 23. Port 6 and Port 7 have input reflection coefficients slightly above −10 dB in the frequency range of 3.0–3.5 GHz. The worst case is |S_77_| = −7.1 dB at 3.5 GHz. As for the results shown in Figure 18, these results are probably above the experimental ones. The mutual coupling was also simulated. It is negligible for any pair of monopoles with different locations. Even for pairs at the same location, it is below −15 dB, as could be expected considering the results shown in Figure 18. The gain results for each port are almost the same as for the dual-linearly polarized monopole, as shown in Figure 20.

To simulate the voltage induced in each port by the electromagnetic radiation present in the environment, a plane electromagnetic wave, with an electric field intensity of 1 V/m, was considered. The direction of incidence was varied in the angles θ (from 0 to 180 degrees) and φ (from 0 to 360 degrees) in 45 degrees steps. Vertical, horizontal, and 45 degrees inclined linear polarizations were studied. In the simulations, each of the 10 monopole ports was terminated with 50 Ω loads, which corresponds to the ideal situation of a perfect impedance match between the receiving antenna and the receiver.

**Figure 24:**
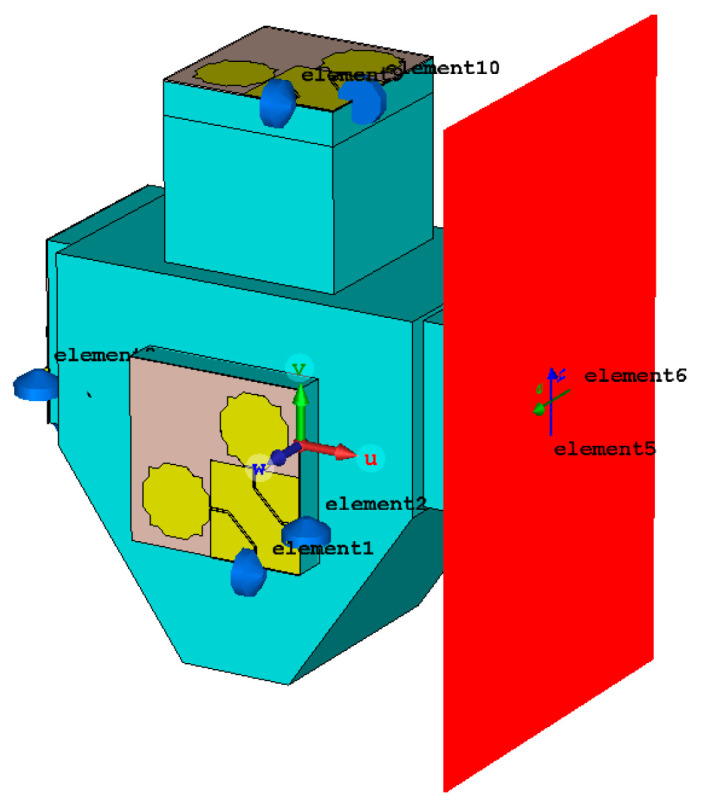
CST model for the example of electromagnetic plane wave incident from (θ = π/2,φ = 0), with horizontal (Z) polarization.

**Figure 25:**
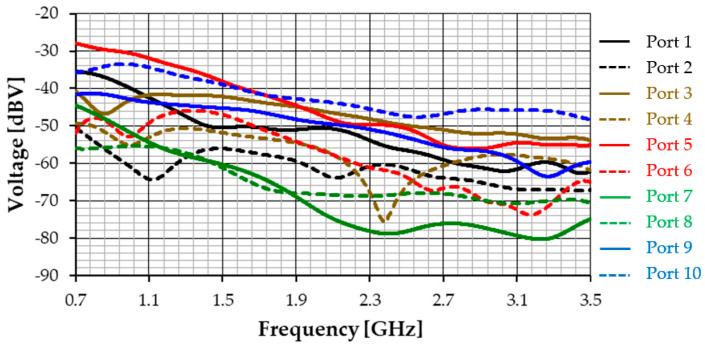
Voltage induced at each port for the example of incidence shown in Figure 24.

As an example, the incidence from θ = 90 degrees to φ = 0 of the plane electromagnetic wave with horizontal polarization is shown in Figure 24. The corresponding amplitude of the voltage induced at each port is shown in Figure 25.

As expected, Port 5 and Port 10, corresponding to the monopoles with line of sight (LoS) and the perfect (Z) polarization match, have the highest induced voltages. The induced voltage curves tend to follow the gain curve shown in Figure 20. Naturally, the curve for Port 6 confirms the complete polarization mismatch. Moreover, Port 7 and Port 8, located on the opposite shoulder and therefore with a non-line of sight (NLoS) situation, have much weaker induced voltages. It is necessary to point out that the interpretation of the results for the induced voltage at each port needs to take into account the complex propagation scenario created by reflections and scattering caused by the metallic shield that covers the user’s torso.

### 4.3. Electromagnetic Field Estimation

The equivalent Thevenin-induced unloaded antenna voltage (V_0_) can be obtained from [10]

(2) $$V_{0}{=E\;h}_{e}\;\sqrt{C_{p}}$$

where E is the amplitude of the incident electric field, h_e_ is the antenna effective height, and C_p_ is the antenna polarization mismatch factor. Assuming a perfect antenna impedance matching the voltage over the 50 Ω load (V_load_) is half of V_0_. It is also known that [10]

(3) $$h_{e}=\lambda \;\sqrt{\frac{G\;R_{a}}{\pi \;Z_{0}}}$$

where λ is the free-space wavelength, G is the antenna gain, R_a_ = 50 Ω is the antenna input resistance, and Z_0_ = 120π Ω is the characteristic impedance of the vacuum. Combining Equations (2) and (3), and assuming C_p_ = 1, the incident electric field amplitude can be estimated as

(4) $$E_{\mathrm{est}}\left(f\right)=\frac{V_{\mathrm{load}}\left(f\right)\;f\;2\pi \;\sqrt{2.4}}{C_{0}\;\sqrt{G\;(f)}}$$

where f is the frequency of the incident electromagnetic plane wave and C_0_ is the light speed in vacuum.

The simplest procedure that can be used to estimate the amplitude of the electric field of the incident plane electromagnetic wave can be summarized as follows:

- Read the voltage at each of the 10 monopole antenna ports and select the highest one. Users will be instructed to move around while reading the voltages to avoid the eventual incidence of local radiation pattern minima.
- Obtain the monopole gain from Figure 20.
- Use Equation (4) to calculate E_est_.

It is assumed that there is a filtering device that selects the frequency band for each measurement. Taking into account the simplifying assumptions used, this simple procedure underestimates the amplitude of the incident electric field. A more accurate estimation can be obtained by implementing a more elaborate procedure and making use of the information available. For instance, we may use the amplitude and phase of the voltages measured at each port, obtain the direction of arrival (DoA) of the incident electromagnetic wave [44], and use, in Equation (3), the specific gain value for that DoA. It is also possible to determine the polarization of the incoming electromagnetic wave. This tradeoff between the complexity of the estimation procedure (associated hardware and software) and the accuracy of the estimation must be evaluated at the final system-level implementation. However, such analysis is out of the scope of this work.

## 5. Conclusions and Future Work

An antenna system based on a wideband modified planar printed octagonal monopole is proposed for integration into a system conceived for the estimation of electromagnetic field radiation in the frequency range of 0.7–3.5 GHz. This frequency range encompasses all the relevant mobile communication standards, namely, 2G, 3G, 4G, and most of the 5G (below 6 GHz) frequency bands. Such a system is envisaged to be integrated into protective vests worn by professional users in their working space environment as part of an intelligent multi-risk protection system.

Initially, the simple straight monopole is completely characterized to serve as a reference. Then, the modified octagonal monopole with CPW feed is proposed to provide the required wideband. A dual-linearly polarized combination of such modified octagonal monopoles is proposed. The experimental macroscopic characterization of a lossy foam is described. A relatively thick layer of this foam is used to mitigate the unwanted effects of the metal shield on the dual-linearly polarized monopole antenna.

Five dual-linearly polarized monopole antennas will be integrated into the user jacket, located on the chest, back, left, and right shoulders and helmet. Based on the knowledge of the monopole antenna gain, the frequency band of the operation, and the maximal induced voltage at the antenna ports, a simple procedure is proposed to estimate the amplitude of the electric field of the incident electromagnetic plane wave. As part of the tradeoff between the complexity and accuracy of the estimation procedure, other strategies may be followed.

Only the proof-of-concept of the antenna system is described in this paper. Therefore, no strict fulfillment of the specifications was imposed, as this is a task to be accomplished in the final stage of the development of the prototype by an industrial partner. Many topics of future work can be represented as requiring specific analysis for the evolution from the proof-of-concept to the industrial prototype, such as the following:

- Use adequate wearable-oriented materials (lossy foam, dielectric textiles, and metal textiles) [45].
- Integration into the vest (superstrate textiles, monopole deformation, and feeding cables).
- Tests with a realistic phantom (reflection coefficient, radiation pattern, efficiency, and gain).
- Uncertainty analysis of the procedure to estimate the amplitude of the electric field of the incident electromagnetic field.
- Extend the analysis up to 6.0 GHz (to include the remaining sub−6 GHz 5G frequency bands).

We are completely aware that alternatives to the single modified octagonal monopole antenna element could have been adopted initially (for instance, the circular, elliptical, or hexagonal monopoles, just to mention canonical geometries). However, we are convinced that using these other monopole geometries in the final antenna system would lead to an equivalent overall performance.

## Figures and Tables

**Table 1:** Values of the geometrical parameters of the modified octagonal monopole.

Parameter	L_s_	L_g_	W_s_ = W_g_	L_p_	L_i_	L_vh_	L_t_	g	a	b
Value [mm]	150	73	100	75	35.4	25	10	1.5	2	0.4

**Table 2:** Port numbering of the antenna system.

Port Number	Monopole Location	Polarization
1	Chest	Horizontal (X)
2	Chest	Vertical (Y)
3	Back	Horizontal (X)
4	Back	Vertical (Y)
5	Left shoulder	Horizontal (Z)
6	Left shoulder	Vertical (Y)
7	Right shoulder	Horizontal (Z)
8	Right shoulder	Vertical (Y)
9	Helmet	Horizontal (X)
10	Helmet	Horizontal (Z)

## Data Availability

The data are available at the request of the corresponding authors.
